# Hemicraniectomy after middle cerebral artery infarction with life-threatening Edema trial (HAMLET). Protocol for a randomised controlled trial of decompressive surgery in space-occupying hemispheric infarction

**DOI:** 10.1186/1745-6215-7-29

**Published:** 2006-09-11

**Authors:** Jeannette Hofmeijer, G Johan Amelink, Ale Algra, Jan van Gijn, Malcolm R Macleod, L Jaap Kappelle, H Bart van der Worp, the HAMLET investigators

**Affiliations:** 1 Departments of Neurology (JH, AA, JvG, LJK, and HBvdW), Neurosurgery (GJA), and Julius Centre for Health Sciences and Primary Care (AA), University Medical Centre Utrecht, PO Box 85500, 3508 GA Utrecht, The Netherlands; 2 School of Molecular and Clinical Medicine, University of Edinburgh, Edinburgh (MMM), UK

## Abstract

**Background:**

Patients with a hemispheric infarct and massive space-occupying brain oedema have a poor prognosis. Despite maximal conservative treatment, the case fatality rate may be as high as 80%, and most survivors are left severely disabled. Non-randomised studies suggest that decompressive surgery reduces mortality substantially and improves functional outcome of survivors. This study is designed to compare the efficacy of decompressive surgery to improve functional outcome with that of conservative treatment in patients with space-occupying supratentorial infarction

**Methods:**

The study design is that of a multi-centre, randomised clinical trial, which will include 112 patients aged between 18 and 60 years with a large hemispheric infarct with space-occupying oedema that leads to a decrease in consciousness. Patients will be randomised to receive either decompressive surgery in combination with medical treatment or best medical treatment alone. Randomisation will be stratified for the intended mode of conservative treatment (intensive care or stroke unit care). The primary outcome measure will be functional outcome, as determined by the score on the modified Rankin Scale, at one year.

## Background

Large cerebral infarcts are commonly associated with variable degrees of brain oedema. In severe cases, this may lead to transtentorial or uncal herniation. Serious oedema formation usually manifests itself between the second and fifth day after stroke onset [1-4]. Brain tissue shifts rather than raised intracranial pressure (ICP) are probably the most likely cause of the initial decrease in consciousness [4,5].

Fatal space-occupying brain oedema is rare, and occurs in 1 to 5% of patients with a supratentorial infarct [6,7]. However, in younger patients with ischaemic stroke, herniation accounts for about half of the deaths in the first month [8]. Patients with a hemispheric infarct and massive space-occupying brain oedema have a poor prognosis: in recent intensive care (IC)-based prospective series, the case fatality rate was about 80%, despite maximal conservative therapy [9,10].

Several conservative treatment strategies have been proposed to limit brain tissue shifts and reduce intracranial pressure, such as sedation with barbiturates or propofol, hyperventilation, and osmotic therapy with glycerol, mannitol, or hypertonic saline hydroxyethyl starch (HES) [11-13]. However, no trials have addressed the efficacy of these therapies to improve clinical outcome [14], and several reports suggest that these are ineffective [9,10,15] or even detrimental [16,17]. The value of ICP monitoring has also not been established. ICP monitoring may reduce iatrogenic errors [18], but is probably not helpful in guiding long-term treatment [5]. Despite this lack of evidence hyperventilation, osmotherapy, and the use of ICP monitors are recommended and used by experts for patients with ischaemic stroke whose condition is deteriorating secondary to oedema formation [11,12,19].

Because of the limitations of medical therapies, there have been proposals for decompressive surgery in patients with neurological deterioration due to large hemispheric infarction and oedema. The rationale of this therapy is to prevent brain tissue shifts and to normalise intracranial pressure, and thereby to preserve cerebral blood flow and to prevent secondary damage [19]. The technique of decompressive surgery is relatively simple and consists of a large hemicraniectomy and a duraplasty. It can be performed in every neurosurgical centre.

Animal studies have shown that decompressive surgery reduces mortality and improves histological and functional outcome [20,21]. Case reports and small retrospective studies have suggested that this intervention lowers mortality without increasing the rate of severely disabled survivors [19,22,23]. These findings were supported by two prospective series. Mortality was reduced from about 80% in controls to 34% and 16% in surgically treated patients, respectively, and poor functional outcome from 95% to 50% [24,25]. Reports from other studies suggest that decompressive surgery is less effective in elderly patients [26,27], and that substantial recovery extends into the second half year and thereafter [28].

Although the above results suggest a very substantial benefit of decompressive surgery, the studies had too many flaws to be readily translated into clinical practice. Most importantly, the groups were not constituted by random selection. The control groups consisted of patients with a significantly higher age, more co-morbidity, and more frequent lesions in the dominant hemisphere than those in the surgical groups [24,25]. In addition, information on functional outcome of the surviving patients was insufficient [24,25].

The aim of the current study is to test if decompressive surgery would improve functional outcome in patients with neurological deterioration due to large hemispheric infarction and oedema, as compared with 'standard' best medical care, on either a stroke unit (SU) or an ICU, in a randomised controlled clinical trial.

## Methods and design

This is a multi-centre, open, randomised treatment trial with masked outcome assessment. Participating centres have adequate experience with the management of acute ischaemic stroke and intensive care treatment of patients with an elevated ICP, and neurosurgical facilities are available on a 24-hours/day basis. The trial protocol has been approved by the medical ethical committees of all participating centers.

### Enrollment criteria

Patients can be enrolled in the study if they have 1. a diagnosis of acute ischaemic stroke in the territory of the middle cerebral artery, with an onset within 96 hours prior to the planned start of the trial treatment. Within this timeframe, it is advised to start treatment as soon as possible; 2. a score on the National Institutes of Health Stroke Scale (NIHSS) [29] ≥ 16 for right-sided lesions or ≥ 21 for left-sided lesions; 3. a gradual decrease in consciousness to a score of 13 or lower on the Glasgow Coma Scale (GCS) for right-sided lesions, or an eye and motor score of 9 or lower for left-sided lesions; 4. hypodensity on CT involving two thirds or more of the territory of the middle cerebral artery (MCA), and space-occupying oedema formation. (displacement of midline structures on CT is not a requirement for inclusion); and 5. age 18 up to and including 60 years. In addition, there must be a possibility to start trial treatment within 3 hours after randomisation, and written informed consent must be obtained from a representative of the patient.

Patients will be excluded from the study if they have 1. ischaemic stroke of the entire cerebral hemisphere (anterior, middle, and posterior cerebral artery territories); 2. a decrease in consciousness at least partially explained by a cause other than oedema formation, such as metabolic disturbances or medication; 3. two fixed dilated pupils; 4. been treated with a thrombolytic agent in the 12 hours preceding randomisation; 5. a known systemic bleeding disorder; 6. a pre-stroke score on the modified Rankin Scale (mRS) [30] of more than 1 or on the Barthel Index (BI) of less than 95 [31], 7. a life expectancy < 3 years, or 8. a serious illness that may confound treatment assessment.

### Informed consent

Written informed consent for this study will be obtained from the patient's authorised representative prior to the performance of any protocol-specific procedure. However, several of these assessments or tests may be performed as part of the patient's routine clinical evaluation (i.e. not specifically performed for this trial). The study will be conducted in accordance with the provisions of the Declaration of Helsinki, as amended in South Africa (1996).

### Treatment allocation

After informed consent is obtained, patients will be randomised to either surgical or conservative treatment. Randomisation will be stratified for the intended mode of conservative treatment (intensive care or stroke unit care; see below). The choice of conservative treatment is left at the discretion of the local investigator, and will usually be the standard mode of treatment in the participating centre. Allocation to treatment will be made via a telephone call to a 24 hour randomisation service. Ideally, each treatment arm will consist of 56 patients. The total number of patients included in the trial will be 112.

### I. decompressive surgery

Decompressive surgery will consist of a large hemicraniectomy and a duraplasty. In summary, a large (reversed) question mark-shaped skin incision based at the ear will be made. A bone flap with a diameter of at least 12 cm (including parts of the frontal, parietal, temporal, and occipital squama) will be removed. Additional temporal bone will be removed so that the floor of the middle cerebral fossa can be explored. The dura will be opened and an augmented dural patch will be inserted. The dura will be fixed at the margins of the craniotomy to prevent epidural bleeding. The temporal muscle and the skin flap will then be re-approximated and secured. Infarcted brain tissue will not be resected. A sensor for registration of intracranial pressure may be inserted. In surviving patients, cranioplasty will be performed after at least six weeks, using the stored bone flap or acrylate. After surgery, patients will be transferred to an IC unit. Anti-oedema therapy as described under 'IIa' may be used but will usually not be necessary.

### IIa. intensive care

Because no mode of IC treatment has been proven superior to the others, treatment options may vary depending on local traditions. However, to improve consistency of treatment between centres, the following treatment modalities are recommended:

*a. Osmotherapy*. Osmotherapy should be started as soon as possible after randomisation. The use of mannitol or glycerol is recommended in a dose sufficient to reach a serum osmolality of 315 to 320 mOsm.

*b. Intubation *and mechanical ventilation. Patients will be intubated if the score on the GCS is lower than or equal to 10, or if there are signs of respiratory insufficiency, such as a pO2 ≤ 60 mm Hg, a pCO2 ≥ 60 mm Hg, or if the airway is compromised. However, earlier intubation is left at discretion of the treating physician. Mechanical ventilation with use of intermittent mandatory or assist/control ventilation will often be sufficient, especially in the early phase.

*c. Hyperventilation*. The use of hyperventilation is discouraged. If hyperventilation is started, it is advised to monitor venous oxygen saturation with jugular bulb oxymetry and to maintain oxygen saturation higher than 50%. If venous oxygen saturation is not monitored, the pCO2 may be reduced to 28 – 32 mm Hg.

*d. ICP monitoring*. Invasive monitoring of the intracranial pressure is left at the discretion of the treating physician. If used, the ICP monitor should preferably be inserted ipsilateral to the side of the infarct.

*e. Sedation*. If mechanical ventilation requires sedation, and in the case of further neurological deterioration, or an uncontrolled increase in ICP, patients may be sedated with propofol. The use of barbiturates is discouraged, because this may reduce cerebral perfusion pressure and does often not lead to a sustained control of ICP. If necessary, muscle relaxants may be used.

*f. Blood pressure control*. In general, spontaneous elevation of blood pressure to a level of 220/120 mm Hg should be accepted. Sustained higher blood pressures can be treated with labetolol or nitroprusside. In case of sustained hypotension or a critical reduction of cerebral perfusion pressure catecholamines may be used.

*g. Elevation of the head *(30°) to optimise venous drainage.

*h. Aim for normothermia*. Antipyretics and a cooling blanket may be used.

*i. Aim for normoglycaemia*. Consider the use of insulin.

IC treatment should be continued at least until day 5 after stroke onset, or until there is clinical or radiological improvement deemed to be sufficient to transfer the patient back to the stroke unit or medium care unit.

### IIb. stroke unit care

The patient will be admitted to a stroke unit or medium care unit and is treated according to local practice, supplemented with the treatment options that can be performed in a medium care unit as described above under 'a'.

Treatment strategy 'I' is the experimental option, strategy 'IIa' is recommended by international experts, and strategy 'IIb' is the most common clinical practice in the Netherlands, and probably also in a significant number of other European countries.

The choice of ancillary care measures, e.g., prophylactic administration of (low-molecular-weight) heparin and the treatment of complicating illnesses, will be at the discretion of the treating physician. However, administration of low-dose aspirin is recommended, whereas treatment with intravenous heparin and haemodilution with plasma expanders are discouraged.

### Outcome assessment

The primary outcome measure will be functional outcome, as determined by the score on the mRS, at one year. Outcome will be dichotomised as 'good' (mRS 0 to 3) or poor (mRS 4 to dead). The score on the mRS will be determined in a standard way independently by three blinded investigators, on the basis of a narrative written by an unblinded independent study nurse, if necessary followed by a consensus meeting. Secondary analyses will be performed in which 'good outcome' will be defined as mRS 0 to 2, and 'poor outcome' as mRS 3 to dead, and with case fatality as the measure of outcome. Other outcome measures will include the scores on the NIHSS, the BI, and the Montgomery and Asberg Depression Rating Scale (MADRS) [32] and quality of life as measured with the SF3633 and with a visual analogue scale (VAS) [33] at one year and at three years. In addition, the mRS, NIHSS, and BI will be determined at 3 and 6 months.

### Data collection

At baseline, the medical history will be assessed and a general and neurological examination will be carried out. The scores on the GCS and NIHSS will be used to assess stroke severity on admission and directly prior to randomisation. Retrospective scoring of the NIHSS on admission is reliable when a published algorithm is used [34]. Date and time of stroke onset and of neurological deterioration (i.e. GCS ≤ 13) will be noted. Laboratory investigations will include a blood cell count, electrolytes, serum glucose, creatinine, and liver enzymes. Brain CT or MRI scans will be performed on admission and prior to randomisation. Lateral displacement of the pineal gland and the septum pellucidum will be measured by the trial co-ordinator.

The GCS, pupillary reflexes, body temperature, and blood pressure will be assessed every 4 hours during the first 4 days after randomisation. If ICP is recorded, ICP and cerebral perfusion pressure (CPP) will be noted at 2-hour intervals. Dose and time of administration of osmotic agents and sedatives, concurrent medication, and neurological and systemic adverse events will be recorded. The GCS, NIHSS, mRS, and BI will be determined at day 14. CT or MRI of the brain will be performed at day 7 ± 2 days for measurement of infarct volume [35,36] and lateral displacement of the pineal gland and septum pellucidum.

At three and six months, the mRS, BI, and NIHSS will be assessed. Serious adverse events and the number of days the patient was admitted on an IC unit, in the hospital, and/or rehabilitation centre or chronic nursing home will be recorded.

At one and three years, the mRS, BI, MADRS, VAS, and SF36 will be assessed by the research fellow or data manager. He/she will perform a standardised evaluation of each patient, including functional status, and will include the obtained information in an essay. Based on this essay, three members of the executive committee, who are blinded to treatment allocation, will determine the scores on the mRS and BI.

### Statistical aspects

In the primary analysis, outcome of all patients in the surgical group will be compared with that of all patients in the conservative treatment group. However, subgroup analyses will be performed based on the mode of conservative treatment (intensive care vs. stroke unit care), side of the lesion, and time of randomisation (within 48 hours versus between 48 and 96 hours). The sample size is based on the desire that in each of the two subgroups the superiority of surgical decompression over conservative treatment can be proved. All primary analyses will be performed on an intention-to-treat basis, but additional on-treatment analyses will also be performed. An interim analysis will be performed after the one-year follow-up of the first 30 patients.

Power calculations are hampered by the scarcity of data on clinical outcome in this patient group. In the non-randomised trial by Rieke et al., which is most comparable to the proposed study regarding patient group and treatment, 95% (20/21) of the patients in the IC treatment group had a poor outcome, compared with 50% (16/32) in the surgically treated group [24]. There are no data on outcome after 'standard' treatment, but this is not likely to be better than IC treatment. Based on an α of 0.05 and a β of 0.20, extrapolation of these data suggests that only 12 patients would have to be included in each group to demonstrate the superiority of surgical decompression over conservative treatment in a randomised trial. Assuming that 60% of the patients in the surgery group will have a poor outcome and 85% of the patients in each of the two conservative treatment subgroups, a group size of 28 patients would suffice to prove the superiority of decompressive surgery (table 1). Results of applying the same assumption to the complete treatment groups are shown in table 2.

**Table 1:** Results expected for each of the two treatment arms assuming 60% poor outcome after surgery and 85% poor outcome after conservative treatment

	surgery n = 28	conservative treatment n = 28
Poor outcome (n)	17	24
RR (95% CI)	0.71 (0.51–0.99)	ref

RR denotes relative risk; CI, confidence interval. Conservative treatment indicates IC or SU treatment.

**Table 2:** Results expected for the complete treatment groups assuming 60% poor outcome after surgery and 85% poor outcome after conservative treatment

	surgery n = 56	conservative treatment n = 56
Poor outcome (n)	34	48
RR (95% CI)	0.71 (0.56–0.90)	ref

RR denotes relative risk; CI, confidence interval.

### Safety

In the conservative treatment group, patients will receive the standard care of the centre to which they have been admitted. For this reason, the components of the conservative treatment strategies will not be considered experimental, and need no detailed explanation in patient information letters.

Decompressive surgery consisting of a hemicraniectomy and a duraplasty may be complicated by the occurrence of local postoperative bleeding. In the two open, prospective studies of decompressive surgery for space-occupying hemispheric infarction, including a total of 63 patients, 3 patients (5%; 95% CI, 0 to 13) experienced an epidural haematoma, one (2%; 95% CI, 0 to 9) a subdural haematoma, and three (5%; 95% CI, 0 to 13) a space-occupying subarachnoid hygroma over the trepanation site. None of these complications led to an additional neurological deficit [9,25].

An adverse event is any unfavourable and unintended sign, symptom, or disease occurring during the follow-up period of the study. Adverse events occurring after randomisation will be recorded on the adverse event page of the CRF. A serious adverse event is defined as any adverse event that results in death, a life-threatening condition, inpatient hospitalisation or prolongation of existing hospitalisation, or persistent or significant disability/incapacity. An important medical event that may not result in one of the above conditions may be considered a serious adverse event when, based upon medical judgement, it may jeopardise the patient and may require medical or surgical intervention to prevent one of the outcomes above.

A reasonably-related adverse event is defined as one that is possibly, probably, or definitely related to the trial treatment. Adverse events that are serious and reasonably related to the trial treatment, and all deaths, require completion of the safety report, which should be faxed to the trial co-ordination centre within 5 working days of observation or notification of the event.

The Data Monitoring Committee performs analyses of the unblinded interim data and formulates recommendations for the Steering Committee on the continuation of the trial. The Data Monitoring Committee may also offer unsolicited recommendations.

### Publication of the trial results

The trial results will be published by the members of the Executive Committee, on behalf of all HAMLET investigators. Before submission of any manuscript all local principal investigators will have the opportunity to comment on the manuscript.

## Discussion

We present the protocol of a randomised clinical trial designed to test the efficacy of decompressive surgery to improve functional outcome in patients with space-occupying hemispheric infarction. Case reports, retrospective studies and two trials have suggested that this intervention lowers mortality without increasing the rate of severely disabled survivors [19,22-25], but this has never been tested in a randomised, controlled clinical trial.

The timing of surgery and the choice of enrolment criteria form the crux of the present trial. It has been suggested that decompressive surgery is most efficacious if performed within the first 24 hours after stroke onset and before the occurrence of clinical signs of herniation [25], but this may carry the risk of inclusion of patients who do not need such an invasive procedure for survival and recovery. In addition, in a systematic review of studies on decompressive surgery in space-occupying hemispheric infarction, the timing of surgery did not affect outcome [27].

In different studies, several clinical and radiological parameters have been identified as independent early predictors of fatal space-occupying oedema formation: nausea and vomiting within 24 hours after stroke onset [37], a systolic blood pressure of 180 mm Hg or more at 12 hours after onset [37], a history of hypertension or heart failure [38], elevated white blood cell count [38], an activity deficit covering the complete MCA territory on SPECT imaging [39], a hypodensity of more than 50% on the initial CT scan [38,37], attenuated corticomedullary contrast on CT [40], involvement of additional vascular territories [38], and volume of diffusion-weighted imaging (DWI) abnormalities of more than 145 ml [41]. In addition, in the Lubeluzole-International-9 trial it was found that the minimum baseline score in patients who ultimately died from brain swelling was 15 for right-sided lesions and 20 for left-sided lesions [37]. Unfortunately, all studies were relatively small and the value of the suggested predictors has not been confirmed in other prospective series. With the proposed timing of therapy and choice of enrolment criteria we expect to largely prevent inclusion of patients who cannot benefit from hemicraniectomy, either because they do not need decompressive surgery since their disease will follow a more benign course, or because of extensive and irreversible damage.

In the Heidelberg studies, complete global aphasia was an exclusion criterion for decompressive surgery, and the majority of patients receiving surgical therapy had a lesion in the right hemisphere [24,25]. In our study, patients with a large infarct in the dominant hemisphere and severe aphasia may be included, as it is unproven that they will fare worse than patients with an infarct in the non-dominant hemisphere. In fact, it has been shown that with the exception of the ability to communicate, the long-term quality of life of patients with left-sided lesions is slightly better than that of patients with a lesion in the right hemisphere [42].

In this trial, a fully blinded evaluation of the functional outcome of patients is virtually impossible, as patients in the decompressive surgery group will be easily recognised by surgical marks. Even in case of follow up by telephone, patients are likely to note the sequels of surgery. However, sufficient blinding will be assured by the indirect assessment of scores on the functional outcome scales, as described above.

The trial started 1 September 2002. As of 1 July 2006, 52 patients were included in seven participating centres in the Netherlands. Two other Dutch centres and seven centres in the UK are expected to join the trial shortly. Other centres that have adequate experience with the management of acute ischaemic stroke and intensive care treatment of patients with an elevated ICP, and that have neurosurgical facilities available on a 24-hours/day basis are welcome to participate.

### The HAMLET investigators

*Executive Committee*: A. Algra, G.J. Amelink, J. van Gijn, J. Hofmeijer (trial coordinator), L.J. Kappelle, M.R. Macleod (UK national coordinator), and H.B. van der Worp (principal investigator). The *Steering Committee *is constituted of the principle investigators of each actively randomising centre (S.de Bruijn, G.J. Luijckx, R. van Oostenbrugge, J. Stam, and J.Th.J. Tans) and of the members of the executive committee. *Data Monitoring Committee*: Y. van der Graaf, P.J. Koudstaal, and A.I.R. Maas. *Advisory Committee*: G.W. van Dijk, W. Hacke, C.J. Kalkman, C.A.F. Tulleken, and C.A.C. Wijman.

## Competing interests

The author(s) declare that they have no competing interests.

## Authors' contributions

*JH *participated in writing the protocol, applying financial support and approval of medical ethical committees, and is concerned with patient recruitment and data management.

*GJA *wrote the surgical parts of the protocol and performs the hemicraniectomy in the surgically treated patients

*AA *wrote the statistical parts of the protocol

*JvG *participated in writing the protocol, and in applying financial support and approval of medical ethical committees.

*MM *is the UK national coordinator and applied for approval of medical ethical committees in the UK.

*LJK *participated in writing the protocol, and applying financial support, and is concerned with patient recruitment.

*HBvdW *wrote the protocol, applied for financial support, applied for approval of medical ethical committees and is concerned with patient recruitment

## Financial support

This study is funded by the Netherlands Heart Foundation (grant number 2002B138).
